# Recurrent pterygo-palatal angiofibroma with intracranial extension: case report

**DOI:** 10.11604/pamj.2020.36.128.22435

**Published:** 2020-06-25

**Authors:** Fatima Zahra Abboud, Moulay Ali Youssoufi, Sofia Zoukal, Touria Bouhafa, Khalid Hassouni

**Affiliations:** 1 Department of Radiation Oncology, University Hospital Hassan II, Fez, Morocco,; 2 Medical Physics Unit, Oncology Hospital, University Hospital Hassan II, Fez, Morocco,; 3 Epidemiology Laboratory of the Faculty of Medicine and Pharmacy of Casablanca, Casablanca, Morocco

**Keywords:** Angiofibroma, pterygo-maxillary, radiotherapy, IMRT

## Abstract

We report the case of a patient with recurrent pterygo-palatal angiofibroma and its treatment. A 21-year-old male patient had a long history of recurrent epistaxis with progressive nasal obstruction. He was diagnosed with an angiofibroma centered in the right pterygo-palatine fossa. Initially, he underwent surgical excision with removal of the entire tumor. The evolution was clinically good with no signs of recurrence on the cervico-facial scan of control (CT). Nine months after, he presented a reappearance of epistaxis. A cervico-facial MRI was performed and showed a recurrence of the tumor process, which this time was considered inextirpable, hence the decision to opt for radiotherapy with intensity modulated radiation therapy (IMRT). He has improved clinically with a clear reduction in tumor mass on CT scan. This technique represents an interesting alternative to overcome anatomical complexity of the region, cover the tumor and preserve the organs at risk.

## Introduction

Pterygo-palatal angiofibroma or juvenile angiofibroma (JA) is a rare vascular and fibrous tumor with an incidence of 0.05-0.5% of all head and neck neoplasm, and affects young males aged within the range of 12-35 years [1]. It can be life-threatening because of its invasion of adjacent structures and the base of the skull with a risk of extension to the cavernous sinus and optic chiasma [2]. Its treatment is not well codified and is mainly surgical. However, other therapeutic methods such as radiotherapy, interventional arteriography, chemotherapy and hormone therapy are of value in the case of a tumor that cannot be removed. We report here an observation of recurrent pterygo-palatal angiofibroma with intracranial extension, and the interest of radiotherapy in this case.

## Patient and observation

**Figure 1:**
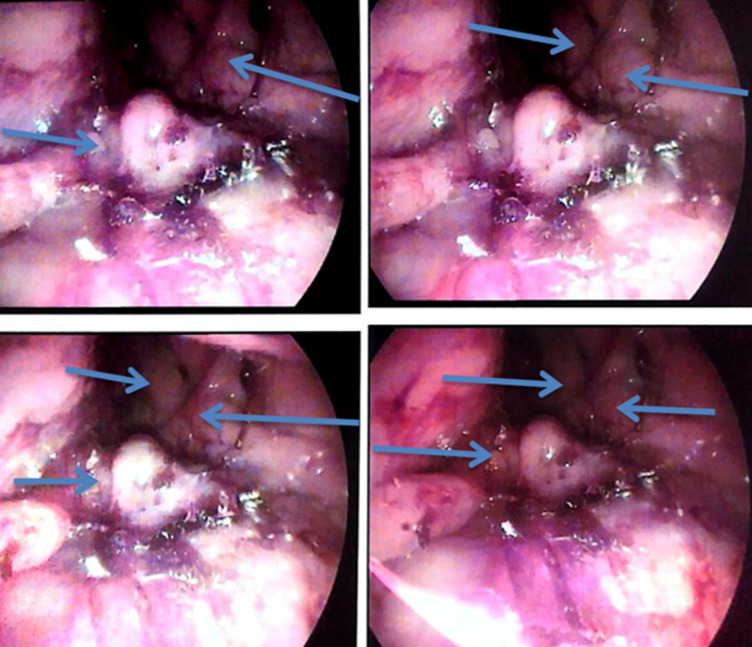
images of the nasofibroscopy performed on the patient following the reappearance of the epistaxis showing the reappearance of the tumor process as indicated by the blue arrows

**Figure 2:**
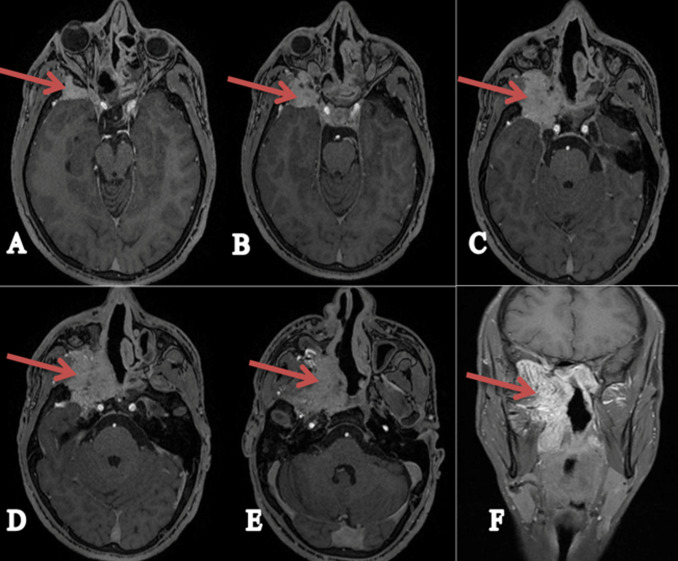
MRI sections in the axial (A+B+C+D+E) + coronal (F) plane sequences T1 with gadolinium injection showing the tumour process on the right side (pterygo-palatine angiofibroma), as shown by the brown arrows

**Figure 3:**
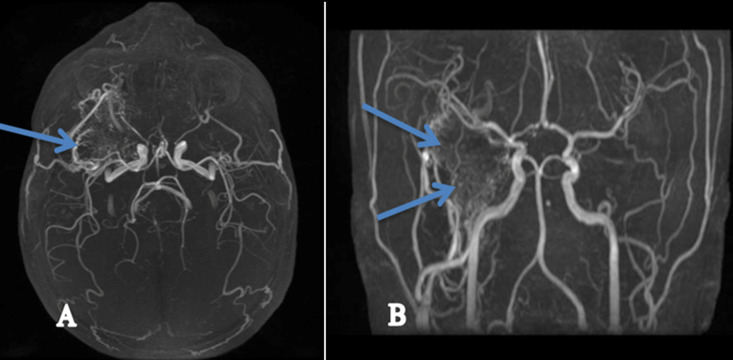
the patient’s angio-MRI showing the tumour blush and feeder pedicles, indicating the hyper-vascularization of this tumour (the angiofibroma) as shown by the blue arrows

**Figure 4:**
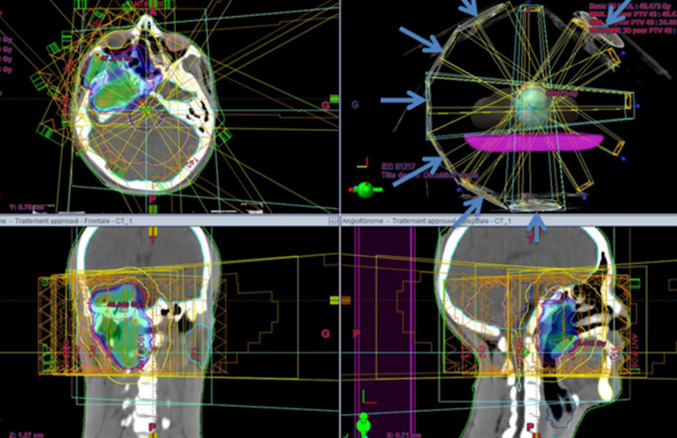
disposition of multiple IMRT beams on the three plans (axial, sagittal and coronal) as shown by the blue arrows

**Figure 5:**
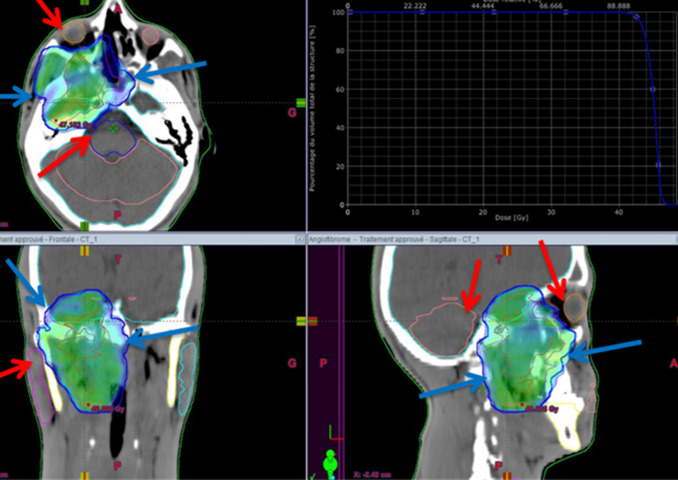
three plans view of the conformity of the prescribed dose to the target volume in blue as shown by the blue arrows and the respect of the organs at risk as shown by the red arrows (right eye, brain stem, cerebellum and right parotid gland)

**Figure 6:**
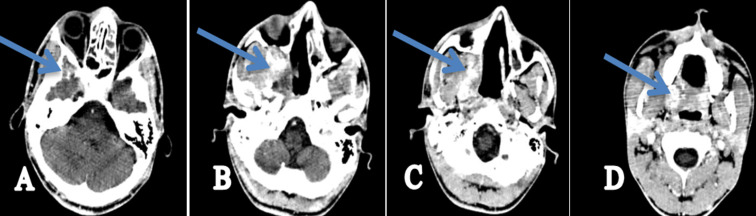
control scan sections (A+B+C+D), in the axial plane showing a marked regression of the tumor process and exophthalmos as shown by the blue arrows

A 21-year-old male patient with a long history of recurrent epistaxis, with onset of progressive right unilateral nasal obstruction, motivated him to consult the Ear, Nose and Throat Department, where he initially benefited from a nasofibroscopy that showed a right pterygo-maxillary fossa process. On physical examination, there was no cervical adenopathy palpable. A cervico-facial CT scan and brain level was performed and showed a hypervascular tumor process centered on the right pterygo-maxillary fossa. The diagnosis of an angiofibroma was confirmed by biopsy with anatomopathological and immunohistochemical analysis. He was operated on with total removal of the tumor. The clinical course was marked by an improvement in the patient's clinical symptomatology with disappearance of epistaxis and nasal obstruction. CT scan of the control facial mass after surgery showed no sign of tumour recurrence. After a free interval of nine months after surgery, the patient presented a reappearance of the epistaxis and obstruction, which this time associated with a notion of right temporal headaches. In addition, it was refractory to usual analgesics, and was associated with decreased visual acuity in the right eye. The patient consulted again in the Ear, Nose and Throat Department. The physical examination found visual acuity at 6/10 of the right eye and 10/10 of the left eye. The fundus of both eyes was normal. There was no cervical lymphadenopathy. A nasofibroscopy done in the patient who showed a recurrence of his tumor process (Figure 1). The exploration was completed by MRI of the facial mass (Figure 2, Figure 3) which revealed the presence of an ethmoidonasal tumour process measuring 70x50x40 mm, heterogeneous with a double cystic and fleshy component, extending into the right maxillary sinus and the homolateral para-pharyngeal space, with extra-axial intracranial extension in the right temporal region. The tumor has been classified V according to the classification of Snyderman *et al*. (Table 1). The patient’s file was discussed in a multidisciplinary consultation meeting and the tumor was judged by the surgeons to be in extirpated. External radiotherapy (RT) was then decided. He was planned for a total dose of 45 Gy of RT in 25 fractions, at a daily dose fraction of 1,8 Gy over 5 weeks using the intensity-modulated radiotherapy (IMRT) technique with fields prescribed to the 100% isodose line, using 6-MV photons. We were able to keep the tolerance doses of organs at risk such as the optic chiasma, optic nerves, retina, pituitary and parotids within normal limits and at the same time deliver the intended dose of radiation to the tumor site (Figure 4, Figure 5). The ophthalmological examination performed at the end of the treatment resulted in an improvement in the visual acuity of the right side measured at 9/10. The patient reported an improvement in clinical symptomatology, with disappearance of epistaxis, nasal obstruction and headache. A CT scan of the facial mass and cerebral area performed at two months after the end of irradiation which showed a clear reduction in tumor mass (Figure 6).

**Table 1:** university of Pittsburgh medical center (UPMC) staging system for angiofibroma

Stage	UPMC Staging System
I	Nasal cavity, medial pterygopalatine fossa
II	Paranasal sinuses, lateral pterygopalatine fossa; no residual vascularity
III	Skull base erosion, orbit, infratemporal fossa; no residual vascularity
IV	Skull base erosion, orbit, infratemporal fossa; residual vascularity
V	Intracranial extension, residual vascularity; M, medial extension; L, lateral extension

## Discussion

JA is a tumor that originates from the posterolateral wall of the arch of the nose, where the pterygoid process of the sphenoid bone and the sphenoid process of the palatal bone constitute the sphenopalatal foramen. From there, several extension patterns occur, mainly towards the nasopharynx, resulting in clinical signs that are often confusing to the uninformed clinician [1]. For example, if the most frequent reason for consultation is epistaxis associated with nasal obstruction or mucopurulent rhinorrhea; other signs of tumor progression may appear, such as exophthalmos, decreased visual acuity, headache or nasosinus pain [2]. Imaging is an essential tool for diagnosis. It includes computed tomography, which is best for assessing extracranial extension, and MRI, which is the test of choice for assessing intracranial extension. Arteriography too is particularly useful for studying these highly vascularized tumors and for guiding embolization [3]. In certain contentious situations, even with very evocative images, biopsy is always justified. It then shows a double vascular and fibrous contingent. The vascular component is made up of thick-walled vessels and vascular lacices, while the fibrous component is made up of a collagen stroma arranged in a dense felt whose essential cellular element is the fibroblast [4]. It confirmed the initial diagnosis in our patient. There is no codified management. However, surgery remains the treatment of choice offering the best results and the best quality of life in the long term for this young population [5]. Each center opted for a conservative surgical technique with divergent results depending on the competence of the surgeon operating, contributing to confusion regarding the surgical approach to be applied, as well as to a multiplicity of staging systems that reflect an evolving and progressive understanding of actual extension patterns [3]. In addition, recurrences have been reported after 6 months of initial treatment, with rates ranging from 3% to 35% in some series [6]. In our patient, the clinical examination and imaging were negative after surgery, but he showed the reappearance of clinical signs such as epistaxis and right nasal obstruction with the reappearance of the tumor process on MRI after 9 months, which leads us to speak of tumor recurrence. When evaluating a recurrent tumor, conservative follow-up for 3 to 6 months is adopted in asymptomatic patients with small to medium sized tumor. However, in cases of invasion of the medial cavernous sinus, when no previous radiotherapy has been given, an IMRT is indicated. The evidence in favor of its use is limited to a few case series with consistent and fairly promising results. On the one hand, because it is generally well tolerated, with a risk of complication that is increased if there is intracranial extension or if a new irradiation is administered. On the other hand, because doses above 36 Gy provide better local control than lower doses of fractionated radiotherapy [7-10]. In the case of our patient, we were confronted with a recurrent pteriopalatine fossa angiofibroma, after a first surgical removal, very aggressive with a decrease in visual acuity of the right eye due to a tumor compressive optic neuropathy. Radiotherapy compensated for the result. At the last MRI check-up after the end of treatment, the tumor had regressed significantly in size. Embolization was not indicated because it could compromise the therapeutic results by preventing vascularization, thus reducing the effectiveness of radiotherapy. The satisfactory result obtained allows us to maintain radiotherapy as a therapeutic option for recurrent petygo-palatal angiofibromas that cannot be removed due to significant intracranial extension.

## Conclusion

Radiotherapy and surgery are the main therapeutic modalities for pterygo-palatal angiofibroma. Although it is not indicated as a first-line treatment, and although pterygo-palatine angiofibroma is a benign tumor, radiotherapy represents an interesting alternative in cases of intracranial extension that cannot be surgically removed.
